# Effectiveness of mid-regional pro-adrenomedullin (MR-proADM) as prognostic marker in COVID-19 critically ill patients: An observational prospective study

**DOI:** 10.1371/journal.pone.0246771

**Published:** 2021-02-08

**Authors:** Giorgia Montrucchio, Gabriele Sales, Francesca Rumbolo, Filippo Palmesino, Vito Fanelli, Rosario Urbino, Claudia Filippini, Giulio Mengozzi, Luca Brazzi

**Affiliations:** 1 Anestesia e Rianimazione 1U, Department of Anesthesia, Intensive Care and Emergency, ‘Città della Salute e della Scienza’ Hospital, Turin, Italy; 2 Department of Surgical Science, University of Turin, Turin, Italy; 3 Clinical Biochemistry Laboratory, Department of Laboratory Medicine, ‘Città della Salute e della Scienza’ Hospital, Turin, Italy; Institut d’Investigacions Biomediques de Barcelona, SPAIN

## Abstract

**Objective:**

To test the effectiveness of mid-regional pro-adrenomedullin (MR-proADM) in comparison to C-reactive protein (CRP), procalcitonin (PCT), D-dimer, lactate dehydrogenase (LDH) in predicting mortality in COVID-19-ICU-patients.

**Methods:**

All consecutive COVID-19 adult patients admitted between March and June 2020 to the ICU of a referral, university hospital in Northern-Italy were enrolled. MR-proADM and routine laboratory test were measured within 48 hours from ICU admission, on day 3, 7 and 14. Survival curves difference with MR-proADM cut-off set to 1.8 nmol/L were tested using log-rank test. Predictive ability was compared using area under the curve and 95% confidence interval of different receiver-operating characteristics curves.

**Results:**

57 patients were enrolled. ICU and overall mortality were 54.4%. At admission, lymphocytopenia was present in 86% of patients; increased D-dimer and CRP levels were found in 84.2% and 87.7% of patients respectively, while PCT values > 0.5 μg/L were observed in 47.4% of patients. MR-proADM, CRP and LDH were significantly different between surviving and non-surviving patients and over time, while PCT, D-dimer and NT-pro-BNP did not show any difference between the groups and over time; lymphocytes were different between surviving and non-surviving patients only. MR-proADM was higher in dying patients (2.65±2.33vs1.18±0.47, p<0.001) and a higher mortality characterized patients with MR-proADM >1.8 nmol/L (p = 0.016). The logistic regression model adjusted for age, gender, cardiovascular disease, diabetes mellitus and PCT values confirmed an odds ratio = 10.3 [95%CI:1.9–53.6] (p = 0.006) for MR-proADM >1.8 nmol/L and = 22.2 [95%CI:1.6–316.9] (p = 0.022) for cardiovascular disease. Overall, MR-proADM had the best predictive ability (AUC = 0.85 [95%CI:0.78–0.90]).

**Conclusions:**

In COVID-19 ICU-patients, MR-proADM seems to have constantly higher values in non-survivor patients and predict mortality more precisely than other biomarkers. Repeated MR-proADM measurement may support a rapid and effective decision-making. Further studies are needed to better explain the mechanisms responsible of the increase in MR-proADM in COVID-19 patients.

## Introduction

From December 2019, when the coronavirus disease (COVID-19) first emerged in Wuhan (China), a total of over 19 million COVID-19 cases and 720,000 deaths have been reported globally [1]. Unfortunately, multiple aspects of the disease, especially regarding identification criteria and early Intensive Care management [2], remain unclear.

As in any context of urgency, finding a biomarker able to identify more severe cases is an objective either desired or difficult to achieve. In COVID-19 pandemic, multiple indicators were able to highlight increased severity, especially if combined together [3], but none have been proved entirely effective.

Since the virus-induced endothelial dysfunction is a possible contributing factor to the evolution of COVID-19 infection [4], the need to evaluate the role of Adrenomedullin (ADM) has been recently highlighted [5]. ADM is a multipotent regulatory peptide with a number of biological activities—vasodilator, positive inotropic, diuretic, natriuretic and bronchodilator—widely expressed throughout the body, including bone, adrenal cortex, kidney, lung, blood vessels and heart. It is upregulated by hypoxia, inflammatory cytokines, bacterial products and shear stress [6]. ADM is even present in pulmonary pneumocytes type 2, smooth muscle cells, neurons and immune cells [7], making it a promising molecule to investigate in relation to ARDS, as realized after cardiac surgery [8].

Due to the fact that ADM measurement is known to be complicated, mid-regional pro-adrenomedullin (MR-proADM) has been proposed as an estimate of ADM [9]. High levels of MR-proADM has been reported in septic patients and particularly its specific prognostic value has been described not only for early diagnosis in the context of Emergency Department (ED), but also for risk stratification and prognosis prediction in Intensive Care Units (ICU) [10–13]. MR-proADM has been hypothesizes to have a role either to identify bacterial infections in ICU patients or to select patient to dismiss towards lower-intensity care wards [14–16].

In the context of severe acute respiratory insufficiency characterizing COVID-19 patients admitted to intensive care units (ICU), we compared the effectiveness of MR-proADM to describe patient’s severity and to predict the mortality with other biomarkers commonly used in sepsis and pneumonia.

## Methods

### Study design and population

It is an observational, prospective, single-centre study conducted between March and June 2020 at the “Città della Salute e della Scienza” University Hospital (Turin, Northern Italy) in the regional referral ICU for the treatment of severe respiratory failure and Extracorporeal Membrane Oxygenation (ECMO) support in combination with two new temporary ICUs created to face the COVID-19 pandemic. The study protocol was approved by the local Ethics Committee and was performed in accordance with the principles set put in the Declaration of Helsinki. The data collection was performed anonymously and the need for consent was waived by the Local Ethics Committee.

All consecutive adult patients requiring ICU admission expected to last longer than 48 hours and suffering from pneumonia by Severe Acute Respiratory Syndrome coronavirus 2 (SARS-CoV-2), confirmed by the real-time polymerase-chain-reaction (RT-PCR) on at least one low respiratory tract specimen [17] were enrolled.

All patients were treated according to internal protocols for the management of patients with severe respiratory insufficiency in combination with the new directions emerged from the recent literature about COVID-19 pneumonia [18, 19].

### Patients’ monitoring: Laboratory exams and imaging

At ICU admission, all patients underwent the assessment of clinical and routine laboratory tests in addition to C-reactive protein (CPR), procalcitonin (PCT), D-dimer, lactate dehydrogenase (LDH), N-terminal prohormone brain natriuretic peptide (NT-pro-BNP), performed according to the methods currently used in our laboratory. These specific exams were repeated at day 3 (after 72 hours), 7 and 14. MR-proADM was measured (see below) during patients’ ICU stay (within 48 hours of from ICU admission), after 72 hours (day 3) and on days 7 and 14.

The Chest X-rays or CT-scans and microbiological cultures of blood, sputum, bronchial aspirate or bronchoalveolar samples were performed based on the intensivist’s judgment to assess the progression of the disease.

### MR-proADM analysis

A sample of blood from an EDTA-containing tube was centrifugated at 4000 rpm for five minutes and then a plasma aliquot was immediately frozen and stored at –80°C. When sufficient samples were collected to complete the capacity of the instrument, the MR-proADM measures were determined by the B.R.A.H.M.S. KRYPTOR compact PLUS (Thermo Fisher Scientific, Hennigsdorf, Germany) automated method using the TRACE (Time-Resolved Amplified Cryptate Emission) technique. The detection limit of the assay was 0.05 nmol/L, while intra- and inter-assay coefficients of variation were under 4% and 11%, respectively.

### Data collection

Patient’s demographic characteristics, clinical history, length of mechanical ventilation and ECMO support, if used, were collected from medical records. Ventilatory free-days (VFDs) were defined as: VFDs  =  0, if subject died within 28 days of mechanical ventilation or if the subject was mechanically ventilated for more than 28 days; VFDs  =  28 − x, if the patient was weaned from ventilation “x” days after its start [20].

All the episodes of ventilator associated pneumonia (VAP) and/or blood stream infection (BSI), except for the SARS-CoV-2 infection, as well as the requirement of vasoactive drugs with the development of septic shock [21], were registered according to the European Centre for Disease Prevention and Control (ECDC) current definitions [22].

Off-label use of anti-inflammatory treatments for COVID-19 (i.e. steroid treatment with intravenous methylprednisolone at any dosage and/or intravenous tocilizumab at 8 mg/kg repeated once) were recorded if administered (yes or not).

All patients were followed-up until the hospital discharge to compute: ICU, 28-day and overall mortality, length of ICU and hospital stay.

### Statistical analyses

Summary data were presented as means and standard deviations or median and interquartile range for continuous variables and as percentages for categorical variables. In univariate analysis, continuous variables were compared using the unpaired t test or Wilcoxon-Mann-Whitney according to distribution type. Categorical variables were compared using Fisher exact test or the Chi-square test, as appropriate.

For survival analysis we used Kaplan Meier method considering a period of 28 days. Log-rank test was used to assess the differences between survival curves considering the MR-proADM cut-off of 1.8 nmol/L reported in community-acquired pneumonia studies either in ED or ICU [23–26].

To control potential confounding effects a logistic regression model adjusted for age, gender, cardiovascular disease, diabetes mellitus and PCT values at baseline was used. Results are presented as Odds Ratio (OR) and confidence interval (CI) at 95% level.

To analyze the time course of biomarkers profiles in the different patient groups, a generalize linear model for repeated measures was used.

Predictive ability of MR-proADM, PCT, CRP, NT-proBNP, LDH and D-dimer to discriminate surviving patients was compared using area under the curve (AUC) and 95% confidence interval (CI) of different receiver-operating characteristics curves (ROC) using DeLong’s test.

All tests were two sided and the level of statistical significance was set at 0.05.

All the analyses were performed with the open-source R (R 3.5.0) and SAS software, version 9.4 (SAS Institute Inc., Cary, NC).

## Results

In the period March 1^st^—April 30^th^, 2020, 57 patients, whose demographic and clinical characteristics are summarized in Table 1, were enrolled. At arrival in our ICU, 38 (66.7%) patients required mechanical ventilation, 9 (15.8%) underwent veno-venous Extracorporeal Membrane Oxygenation (vv-ECMO) and 13 (22.8%) were already affected by a major bacterial super-infection. The Sequential Organ Failure Assessment (SOFA) score at admission in our ICU was 7 (IQR 4–10), median between survivor and non-survivor 4 (IQR 3–7) and 9 (IQR 6–12) respectively (p value 0.002). Thirty-one (54.4%) patients developed new bacterial co-infections within 21 days in ICU, presented as VAP, BSI or VAP combined with BSI (38.6%, 36.8% and 22.8%, respectively). Septic shock occurred in 33.3% of patients.

**Table 1:** Clinical characteristics and outcomes.

**Clinical characteristics**	**Overall N = 57**	**Survivors N = 26**	**Non-survivors N = 31**	***p value***
Age, years, *median (IQR)*	64 (54–71)	59 (53–67)	67 (56–74)	***0*.*040***
Gender, male, *n (%)*	50 (87.7)	22 (84.6)	28 (90.3)	*0*.*513*
Patients transferred from other ICUs, *n (%)*	34 (59.6)	15 (57.7)	19 (61.3)	*0*.*783*
Patients with underlying comorbidities, *n (%)*	44 (77.2)	18 (69.2)	26 (83.9)	*0*.*189*
Obesity, *n (%)*	25 (43.9)	11 (42.3)	14 (45.2)	*0*.*829*
Hypertension, *n (%)*	31 (54.4)	13 (50.0)	18 (58.1)	*0*.*543*
Cardiovascular disease, *n (%)*	11 (19.3)	1 (3.8)	10 (32.3)	***0*.*008***
Chronic lung disease, *n (%)*	2 (3.5)	2 (7.7)	4 (12.9)	*1*.*000*
Acute smoker, *n (%)*	6 (10.5)	3 (11.5)	3 (9.7)	*1*.*000*
Diabetes mellitus, *n (%)*	11 (19.3)	2 (7.7)	9 (29.0)	*0*.*051*
Invasive mechanical ventilation at arrival, *n (%)*	38 (66.7)	15 (57.7)	23 (74.2)	*0*.*188*
ECMO at arrival, *n (%)*	9 (15.8)	4 (15.4)	5 (16.1)	*1*.*000*
Superinfection at arrival, *n (%)*	13 (22.8)	3 (11.5)	10 (32.3)	*0*.*111*
Steroids, *n (%)*	21 (36.8)	8 (30.8)	13 (41.9)	*0*.*474*
Tocilizumab, *n (%)*	26 (45.6)	15 (57.7)	11 (35.5)	*0*.*094*
**Outcomes**	**Overall N = 57**	**Survivors N = 26**	**Non-Survivors N = 31**	***p value***
28-day mortality, *n (%)*	27 (47.4)			
Overall mortality, *n (%)*	31 (54.4)			
VFDs at day 28 in ICU, *median (IQR)*	4 (0–2)	14 (0–20)	0 (0–0)	***< 0*.*001***
ICU LOS, days, *median (IQR)*	15 (8–26)	9 (6–25)	20 (11–28)	*0*.*054*
Hospital LOS, days, *median (IQR)*	24 (17–32)	28 (20–35)	20 (12–30)	***0*.*006***
Superinfection within 21 days in ICU, *n (%)*	31 (54.4)	10 (38.5)	21 (67.7)	***0*.*027***
VAP, *n (%)*	22 (38.6)	6 (23.1)	16 (51.6)	***0*.*028***
BSI, *n (%)*	21 (36.8)	9 (34.6)	12 (38.7)	*0*.*750*
Septic shock, *n (%)*	19 (33.3)	4 (15.4)	15 (48.4)	***0*.*011***

*List of abbreviations*: IQR: interquartile range; ICU: intensive care unit; ECMO: extracorporeal membrane oxygenation; VFDs: ventilatory free days; LOS: length of stay; VAP: ventilatory acquired pneumoniae; BSI: blood stream infection.

ICU and overall mortality were 54.4% (no patient died after ICU discharge); 22 (38.6%) patients were discharged to medical wards while four patients were still in ICU on June 30^th^, 2020. Median ICU and hospital length of stay were 15 and 24 days, respectively. The incidence of superinfection and septic shock were lower in survivors (p-value 0.027 and 0.011, respectively) (Table 1).

Within the first 24 hours, lymphocytopenia (defined as lymphocytes <1.50 × 109/L) was present in 86% of patients and was severe (lymphocytes <1.0 × 109/L) in 56.1%; increased D-dimer (defined as > 500 μg/L) and CRP levels (> 10 mg/L) were found in 84.2% and 87.7% of patients respectively, while PCT values higher than 0.5 μg/L were observed in 47.4% (Table 2).

**Table 2:** Laboratory parameters at baseline.

	*Laboratory range*	Overall N = 57	Survivors N = 26	Non-Survivors N = 31	*p value*
WBC, *cell x10*^*9*^*/L*	*4*.*00–10*.*00*	12.85 ± 7.88	10.14 ± 6.27	15.13 ± 8.44	***0*.*016***
Lymphocytes, *cell x10*^*9*^*/L*	*1*.*00–4*.*00*	1.03 ± 1.11	1.27 ± 1.53	0.82 ± 0.47	*0*.*690*
D-dimer, *ng/mL*	*< 540*	10872 ± 21561	10714 ± 24570	11001 ± 19209	*0*.*895*
LDH, *UI/L*	*250–450*	879 ± 345	749 ± 196	923 ± 295	***0*.*007***
NT-proBNP, *pg/mL*	*< 300*	2988 ± 12102	514 ± 760	5153 ± 16407	***0*.*009***
PCT, *μg/L*	*<0*.*5*	3.34 ± 8.15	1.37 ± 3.48	4.93 ± 10.30	***0*.*050***
CRP, *mg/L*	*< 5*.*0*	131.8 ± 104.6	115.5 ± 105.3	145.5 ± 103.7	*0*.*208*

The parameters corresponded to the first available measures performed within 24 hours from ICU admission. Values are expressed as mean ± standard deviation (SD).

*List of abbreviations*: WBC: white blood cell; LDH: lactate dehydrogenase; NT-proBNP: N-terminal prohormone of brain natriuretic peptide; PCT: procalcitonin; CRP: C-reactive protein.

**Fig 1:**
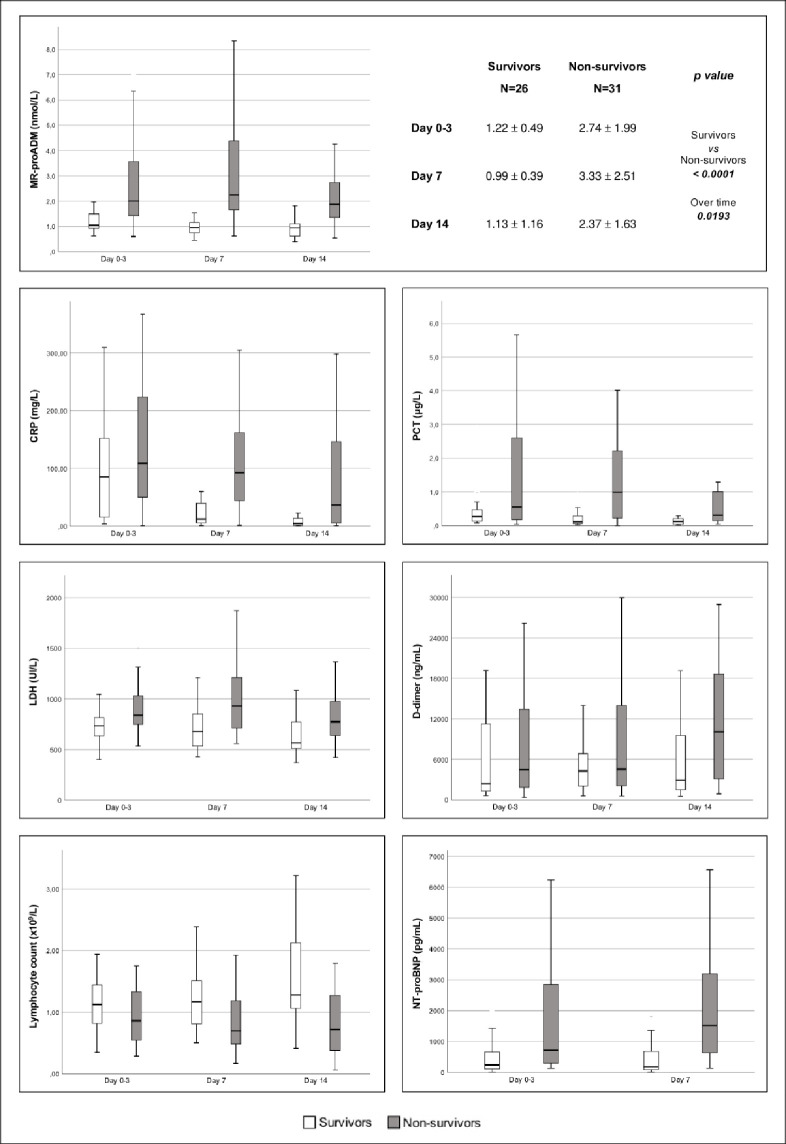
Trends of MR-proADM, CPR, PCT, LDH, D-dimer, lymphocytes and NT-proBNP from ICU admission (day 0) to day 14 in survivor (white) and non-survivor (grey) patients. Time dependent analysis of MR-proADM, CRP and LDH were significantly different between survivors and non-survivors (p-value <0.0001, 0.04 and 0.0253, respectively) and over the time (p-value 0.0193, 0.0003 and 0.0095, respectively), while lymphocytes differed only between survivors and non-survivors (p-value 0.0463). List of abbreviations: MR-proADM: mid-regional pro-adrenomedullin; CRP: C-reactive protein; LDH: lactate dehydrogenase; PCT: procalcitonin; NT-proBNP: N-terminal prohormone of brain natriuretic peptide.

MR-proADM, CRP and LDH were significantly different between surviving and non-surviving patients and over time, while PCT, D-dimer and NT-pro-BNP did not show any difference between the groups and over time; the lymphocytes count was different between surviving and non-surviving patients only (Fig 1).

**Fig 2:**
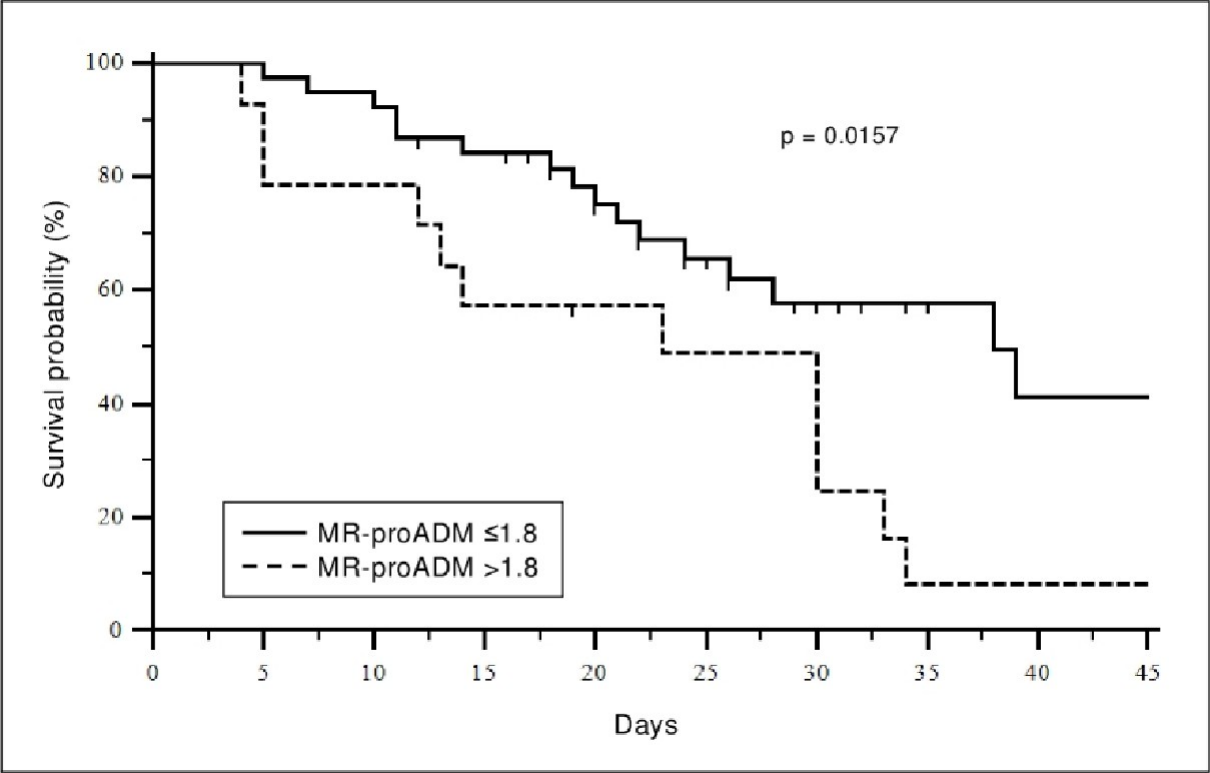
Kaplan-Meier survival curve. Stratification of patients with mid-regional pro-adrenomedullin (MR-proADM) levels greater or less than 1.8 nmol/L at ICU admission.

MR-proADM value at 48 hours from ICU admission (defined as “predictive value”) was significantly higher in dying patients (2.65±2.33vs1.18±0.47, p-value <0.001) and a higher mortality characterized patients presenting a predictive value of MR-proADM exceeding the cut-off of 1.8 nmol/L (p value 0.016) (Fig 2).

This result was confirmed by the logistic regression model adjusted for age, gender, cardiovascular disease, diabetes mellitus and PCT values at baseline which confirmed a statistically significant odds ratio equal to 10.3 [95%CI:1.9–53.6] (p = 0.006) for MR-proADM values higher than 1.8 nmol/L and equal to 22.2 [95%CI:1.6–316.9] (p = 0.022) for cardiovascular disease (Table 3).

**Table 3:** Multivariate logistic regression analysis for mortality.

	Odds Ratio	95% CI	*p value*
Age	1.004	0.932–1.083	*0*.*9121*
Gender, male	3.020	0.390–23.393	*0*.*2899*
Cardiovascular disease	**22.206**	**1.556–316.960**	***0*.*0223***
Diabetes mellitus	7.481	0.964–58.062	*0*.*0543*
PCT values at baseline	1.113	0.945–1.312	*0*.*1998*
MR-proADM >1.8 nmol/L	**10.274**	**1.970–53.578**	***0*.*0057***

*List of abbreviations*: CI: confidence interval; PCT: procalcitonin; MR-proADM: mid-regional pro-adrenomedullin.

**Fig 3:**
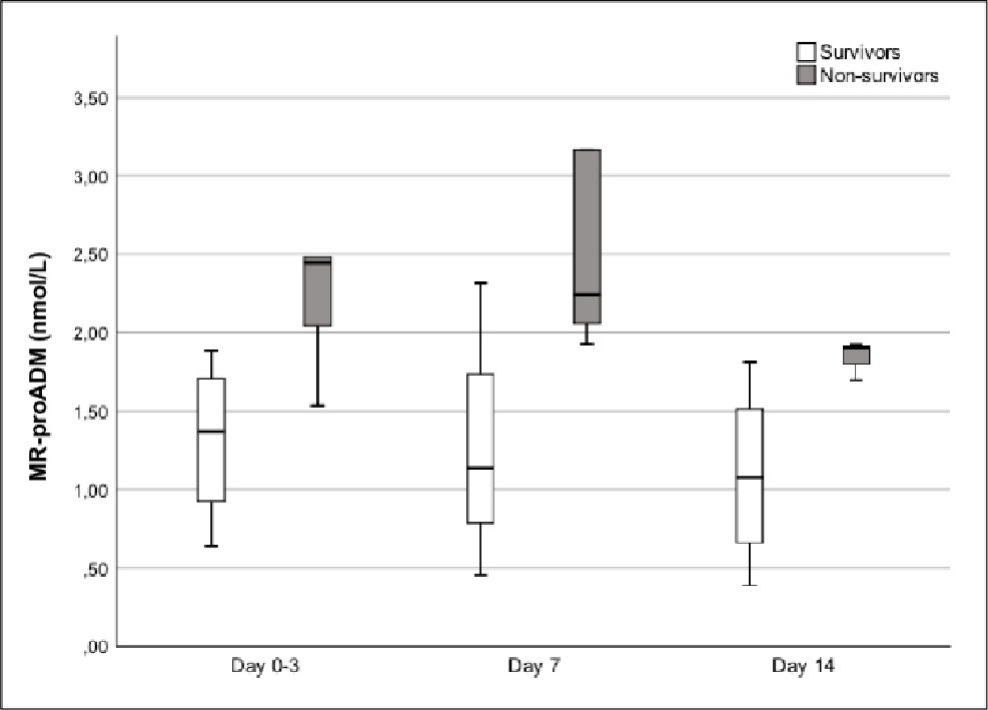
Trends of mid-regional pro-adrenomedullin (MR-proADM) from ICU admission (day 0) to day 14 in survivor (white) and non-survivor (grey) patients who required VV-ECMO. Time dependent analysis of MR-proADM was significantly different between survivors and non-survivors (p-value 0.042) but it was not over the time (p-value 0.205).

MR-proADM trend analysis in the subgroup of patients undergoing vv-ECMO evidenced a statistically significant difference between surviving and non-surviving patients (p = 0.046) but not over time (p = 0.205) (Fig 3).

**Fig 4:**
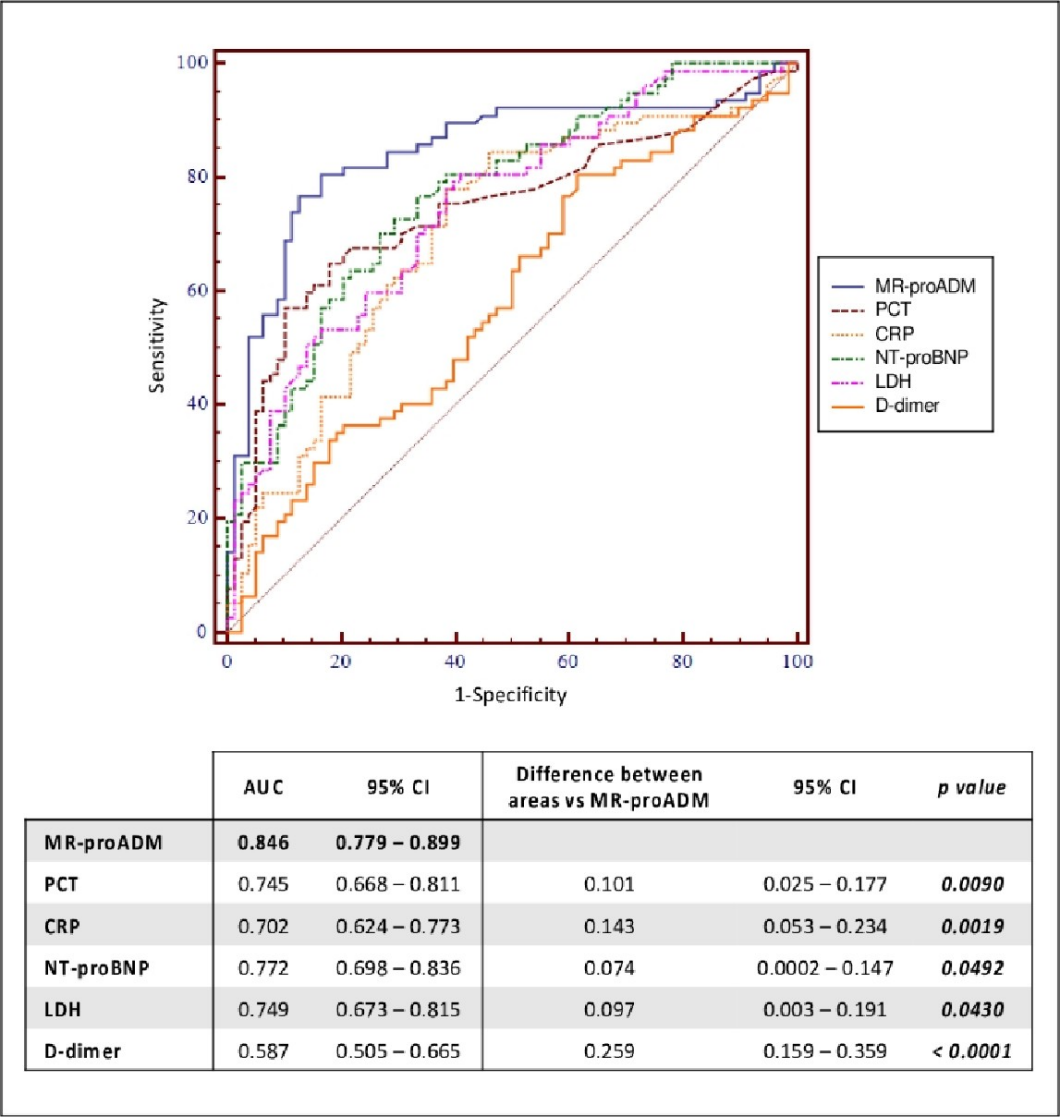
ROC curves of MR-proADM, PCT, CRP, NT-proBNP, LDH and D-dimer and their comparison for predicting mortality. List of abbreviations: MR-proADM: mid-regional pro-adrenomedullin; PCT: procalcitonin; CRP: C-reactive protein; NT-proBNP: N-terminal prohormone of brain natriuretic peptide; LDH: lactate dehydrogenase; AUC: area under the curve; CI: confidence interval.

Overall, MR-proADM was found to have the best predictive ability compared to other biomarkers as shown in Fig 4 (AUC = 0.85 [95%CI:0.78–0.90]). The combination of different biomarkers (MR-proADM+PCT: AUC = 0.85 [95%CI:0.79–0.90]; MR-proADM+CRP: AUC = 0.84 [95%CI:0.78–0.89]) was unable to reach a better statistically significant result.

## Discussion

The present prospective observational study evidenced that MR-proADM, measured within 48 hours from admission, was significantly higher in patients with worse outcome and that a higher mortality characterized patients presenting a predictive value of MR-proADM exceeding the cut-off of 1.8 nmol/L [23–26]. A result maintaining its statistical significance even in a multivariate logistic regression model including age, gender, cardiovascular disease, diabetes mellitus and PCT values at admission as possible confounding. Moreover, its ability to discriminate surviving patients was found to be better than all other biomarkers usually measured in ICU.

Finding a biomarker able to identify patients with worst outcome has always been a challenge, especially in ICU, but emerged as a priority in the context of the pandemic induced by COVID-19 leading even the most advanced health systems to face the problem of the limited health resources available.

MR-proADM appears to be a biomarker with a strong prognostic value. This finding is even supported by a series of experimental evidences attributing to ADM a major role in the regulation of vascular permeability and endothelial barrier [27], on inflammation mediators and microcirculation [28]. All mechanisms certainly playing a role in the development of organ failures characterizing the pathology induced by SARS-CoV2.

To the best of our knowledge, none previously compared sequential measurements of conventional biomarkers such as PCT, C-RP, and other laboratory tests such as lymphocytes, NT-pro-BNP, LDH with MR-proADM values in a cohort of COVID-19 critically ill patients admitted in ICU.

The choice to study the progress of some biomarkers already in use in clinical practice in our patients was taken in accordance with the evidences from the existing literature at the beginning of the pandemic. It is in fact known that a number of laboratory parameters are altered in COVID-19 patients and that some of these alterations, such as the decrease in lymphocyte count [29] and increase in LDH, D-dimer, CRP [30, 31] could be considered predictors of adverse clinical outcomes.

In the population we studied, we observed lymphopenia and an increase in CRP and LDH. NT-proBNP showed a high value at baseline, not confirmed by the values of the trend over time.

Although serum procalcitonin levels are generally normal in patients with viral infections, we observed that these increased in about half of our cases and continued to remain higher than normal during ICU stay. It is reasonable that this increase reflects the occurrence of bacterial superinfection possibly contributing to unfavorable outcomes [32].

MR-proADM ROC analysis showed that this biomarker has significantly greater prediction skill. It is therefore reasonable to hypothesize that MR-proADM is the best biomarker to predict outcome of patients either at admission or during ICU stay. It follows that, as found in sepsis and septic shock [10, 16], the inclusion of MR-proADM monitoring into an early clinical management protocol may support diagnostic intervention and facilitate the choice of the most appropriate treatment in case of organ dysfunctions.

The rise of MR-proADM, resulting from a dose-response mechanism induced by the host-pathogen interaction, appears to occur in the initial phase of pathogen recognition which means, in the case of COVID-19, at the time of hospital admission or even before. However, it is interesting to note that the increase tends to persist over the days in line with the persistence of the disease, underlying the additional value of this biomarker in predicting patient’s outcome. Low values, on the other hand, seems to characterize patients with a shorter ICU length of stay and a lower mortality. It follows that MR-proADM could even be an interesting biomarker to monitor the disease progression even if this is outside from the objectives of the present study.

So far, a predictive value of individual MR-proADM measurements has been found in ischemic and congestive heart disease [33, 34], after cardiac surgery [8], in sepsis [35], in pneumoniae [13, 36] and in other ARDS-related conditions [37]. Despite the low number of cases affected by serious viral pathologies included in previous studies [14, 38], we believe that a similar effect can be hypothesized in the context of cohorts of COVID-19 patients. It is hence possible, as already proposed in the context of sepsis [16], that the use of this biomarker could help either to anticipate the escalation of therapy in patients at risk of treatment failure or to suggest a faster discharge of patients with a low risk of unfavorable evolution.

Particularly interesting is the observation that MR-proADM trend, in our small subpopulation of patients treated with ECMO, is significantly different between surviving and non-surviving patients. This in spite of the typical inflammatory response proved to be associated with the use of extracorporeal circulation [39]. This observation certainly deserves further investigation in larger cohorts of patients treated with extracorporeal circulation and monitored by MR-proADM.

A further aspect to discuss regards the identification of an appropriate cut-off of MR-proADM. Although Krintus et al. reported normality values ranging between 0.21 (0.19–0.23) and 0.57 nmol/L [9], the literature is not univocal in defining a single pathological cut-off. There are evidences, mainly related to sepsis, septic shock, and community-acquired pneumonia (CAP) proposing values ranging between 0.9 and 5.19 nmol/L [36, 40] or more. Considering COVID 19 as a severe community pneumonia, we chose the cut-off 1.8 nmol/L, suggested by important studies on pneumonia [23–26]. The same cut-off is suggested concerning the role of MR-proADM in predicting mortality in patients with sepsis or septic shock [41–43]. Of course, the complexity of this new and only partially known infection makes it difficult to classify, and probably different values (as proposed in the context of septic shock or multi-organ failure) could be adequate, considering that the cut-off may change accordingly to the outcome investigated [13].

We included a population with clinical characteristics that are comparable with others Italian experience [19, 44]: a high proportion required mechanical ventilation at ICU admission (66.7%); a median age of 64 years, with a high preponderance of the male sex (87.7%), and at least one comorbidity in the 77.2%. Cardiovascular disease (other than hypertension) represented the most important comorbidity, with a statistically significant impact on outcome (p value 0.008) even confirmed by the multivariate analysis (OR: 22.2 [IC:1.6–316.9]). This in line with a large-scale study reporting that cardiovascular disease was a major risk factor for fatality of COVID-19 patients [45].

We even observed that 22.8% of our patients presented superinfection at arrival and 54.4% within 21 days in ICU. This aspect deserves particular attention. Bacterial and fungal infections are in fact common complications of viral pneumonia, especially in critically ill patients. Therefore, even among a wide number of articles reporting on COVID-19 clinical data, only a few have reported secondary infection, mostly without detailed pathogens. Some reported a low proportion of superinfections in hospitalized patients (7–8%) that rose to 14% in ICU, less than in previous influenza pandemics [46]. Others reported that secondary infections were identified in 5%-44% of ICU patients with COVID-19, being bacterial or fungal pneumonia and bloodstream the most frequent infections [47], as in our cohort (VAP 38.6% and BSI 36.8%), where the total number of superinfections is higher overall. Interestingly, we found that 33.3% of our patients developed septic shock due to superinfections. These data appear more representative of a population of critically ill patients, with the need for invasive support, prolonged hospitalization, and subjected to repeated, broad-spectrum antibiotic therapies.

Concerning mortality, our data are slightly higher than the values reported in the literature (54.4%). In fact, in a recent metanalysis on 24 observational studies, in patients with completed ICU admissions with COVID‐19 infection, combined ICU mortality (95%CI) was 41.6% (34.0–49.7%) [48]. To note, the in‐ICU mortality from COVID‐19 is far higher than usually seen in ICU admissions with other viral pneumonias. Importantly, the mortality from completed episodes of ICU differs considerably from the crude mortality rates in some early reports. For instance, in the Lombardy experience, ICU mortality was 26% as of March 25, 2020 [44] but rose to 48.7% in ICU and 53.4% in hospital as of May 30, 2020 [19]. It is undoubtedly true that our population had particularly severity conditions at admission, as confirmed by the high percentage of patients transferred from other hospitals (59.6%); of patients undergoing VV-ECMO (15.8%) or suffering from superinfection (54.4%) and septic shock (33.3%).

### Limits

Our study has some limits including a small sample size and a different ICU admission times explained by the fact that our hospital, as a regional reference center, received particularly serious patients as secondary admission. Furthermore, no severity scores other than SOFA are available as the context of the pandemic made data collection particularly difficult. Again, due to the emergency context, it was not possible to have a comparison population and therefore our cohort mainly includes mechanically ventilated patients. Finally, the role of possible impact of confounding factors such as bacterial superinfections, cardiovascular disfunction and renal failure cannot be clearly defined in this first cohort of patients.

## Conclusions

MR-proADM is a new biomarker that seems to be able to effectively measure endothelial damage. Its increased plasma levels indicate severity and worse prognosis in CAP, sepsis, ARDS, perioperative care. The results of this study seem to suggest that, even in the context of the COVID-19 pandemic, MR-proADM have constantly higher values in dying patients and seems able to predict mortality better than other biomarkers. Repeated MR-proADM measurement may hence support a rapid and effective decision-making.

Given that many aspects of COVID-19-induced disease are still unclear, we believe that further studies are needed to better explain the mechanisms involved in the increase in MR-proADM in patients with SARS-CoV-2 and in particular its relationship with multi-organ failure and superinfections.

## List of abbreviations

ADM: adrenomedullin
AUC: area under the curve
BSI: blood stream infection
CAP: community-acquired pneumonia
CI: confidence interval
COVID-19: coronavirus disease 2019
CPR: C-reactive protein
ECDC: European Centre for Disease Prevention and Control
ECMO: Extracorporeal Membrane Oxygenation
ED: Emergency Department
ICU: Intensive Care Units
IQR: interquartile range
LDH: lactate dehydrogenase
LOS: length of stay
MR-proADM: mid-regional pro-adrenomedullin
NT-pro-BNP: N-terminal prohormone brain natriuretic peptide
OR: Odds Ratio
PCT: procalcitonin
ROC: receiver-operating characteristics curves
RT-PCR: real-time polymerase-chain-reaction
SARS-CoV-2: severe acute respiratory syndrome coronavirus 2
SOFA: Sequential Organ Failure Assessment
VAP: ventilator associated pneumonia
VFDs: Ventilatory free-days
vv-ECMO: veno-venous Extracorporeal Membrane Oxygenation
WBC: white blood cell
